# Posture and Locomotion Coupling: A Target for Rehabilitation Interventions in Persons with Parkinson's Disease

**DOI:** 10.1155/2012/754186

**Published:** 2012-01-09

**Authors:** Marie-Laure Mille, Robert A. Creath, Michelle G. Prettyman, Marjorie Johnson Hilliard, Katherine M. Martinez, Colum D. MacKinnon, Mark W. Rogers

**Affiliations:** ^1^UFRS STAPS, Université du Sud Toulon-Var, La Garde 83957, France; ^2^ISM, Aix-Marseille University, Marseille 13288, France; ^3^UMR 6233, CNRS, Marseille 13288, France; ^4^Department of Physical Therapy and Human Movement Sciences, Feinberg School of Medicine, Northwestern University, Chicago, IL 60611, USA; ^5^Department of Physical Therapy and Rehabilitation Science, University of Maryland School of Medicine, Baltimore, MD 21201, USA

## Abstract

Disorders of posture, balance, and gait are debilitating motor manifestations of advancing Parkinson's disease requiring rehabilitation intervention. These problems often reflect difficulties with coupling or sequencing posture and locomotion during complex whole body movements linked with falls. Considerable progress has been made with demonstrating the effectiveness of exercise interventions for individuals with Parkinson's disease. However, gaps remain in the evidence base for specific interventions and the optimal content of exercise interventions. Using a conceptual theoretical framework and experimental findings, this perspective and review advances the viewpoint that rehabilitation interventions focused on separate or isolated components of posture, balance, or gait may limit the effectiveness of current clinical practices. It is argued that treatment effectiveness may be improved by directly targeting posture and locomotion coupling problems as causal factors contributing to balance and gait dysfunction. This approach may help advance current clinical practice and improve outcomes in rehabilitation for persons with Parkinson's disease.

*“. . .postural activity should be regarded as a function in its own right and not merely as a component of movement. . .”*

James Purdon Martin

## 1. Introduction

Disorders of posture, balance, and gait associated with falls and related injuries are among the most debilitating symptoms of advancing Parkinson's disease (PD). In his seminal studies of these clinical sequelae in patients with postencephalitic Parkinsonism entitled *The Basal Ganglia and Posture *published over forty years ago, the British neurologist James Purdon Martin documented with great detail the disorders of postural fixation, righting reactions, and locomotion that similarly often accompany the progression of idiopathic PD [1]. His observations on facilitating functional movements—by gently rocking patients prior to chair rising or gait initiation, by placing bold transverse lines on the walking surface, or through the use of vision to compensate for proprioceptive deficits—influenced the development of current rehabilitation approaches. Purdon Martin later summarized his perspectives on the integration of posture and movement by emphasizing that “…postural activity-should be regarded as a function in its own right and not merely as a component of movement…” [2]. He concluded that all of the conditions on which stepping and consequently locomotion depend (e.g., antigravity support of the body, equilibrium, propulsion) are postural in nature and that even stepping, which prevents the body from falling forward, serves a postural function.

Although neurophysiological studies in both quadrupeds and humans have indicated that the control of posture and locomotion is interdependent at many levels of the central nervous system (CNS) encompassing multiple supraspinal and spinal networks [3–6], the ways by which locomotion may be affected by prevailing postural conditions are not well understood. This is particularly relevant to the problems of postural instability and gait disorders that accompany advancing PD. Such problems are especially evident when patients with PD attempt to perform complex whole body posture and locomotion sequences during functional activities such as gait initiation, sit-to-stand and other transfers, turning while standing and walking, and in ongoing gait. During such tasks, hesitation delays or “freezing” episodes are frequently observed, and the normal pattern of spatial and temporal sequencing between postural and locomotor elements of the task is either absent or disrupted [7]. Thus, the question arises as to whether or not at least some of the difficulties with locomotion experienced by individuals with PD are attributable to a dysfunction of the neuronal networks that mediate the coupling between posture and locomotion. This issue appears to have important implications for current rehabilitative interventions. For example, current physical therapy and rehabilitation interventions for posture, balance, and gait disorders in PD mainly focus on separate aspects of the problems such as posture and balance training [8, 9] or gait training [10–14]. However, impaired *coupling *between posture and locomotion could contribute to gait and mobility disorders, due not only to biomechanical limitations but also to adaptive changes in neural control. For example, De Nunzio et al. have recently demonstrated that alternate rhythmic vibration during quiet stance of bilateral paraspinal muscles affecting trunk posture produced a cyclic transfer of the center of pressure mimicking the one accompanying body progression during walking [15]. When vibration was applied to the trunk musculature during gait, walking velocity, cadence, and stride length increased in both patients with PD and controls [16]. In contrast, no effects on gait were observed when leg muscles (soleus and tibialis anterior) were similarly vibrated. Since the paraspinal muscles contralateral to the single support stance leg play a role in the stabilization of trunk posture during stance, these results suggest that proprioceptive feedback from postural muscles can be used to improve the coupling of posture and locomotion elements of the gait cycle, thus facilitating performance of the task in people with PD.

The purpose of this perspective and review is to present a framework with supportive research findings to advance the viewpoint that focusing rehabilitation interventions on individual or isolated components of posture, balance, or gait disorders in persons with PD should be reevaluated. Instead, it is argued that the emphasis in intervention approaches ought to be shifted towards therapeutic training programs that directly target impairments in posture and locomotion coupling as a causal factor contributing to balance and gait dysfunction.

## 2. Conceptual and Theoretical Model

The difficulties with performing complex whole body posture and locomotion sequences during functional activities such as gait initiation [17–21], turning [22, 23], sit-to-stand [24, 25], and ongoing walking [16, 26] are commonly accompanied by timing delays in the coupling between postural movements of the body segments and the goal-intended locomotion action (e.g., stepping release, step redirection change in turning while walking, seat-off in chair rise, continuous walking). The conceptual and theoretical framework for developing intervention approaches that target impairments in posture and locomotion coupling is illustrated by focusing on the initiation of gait. During gait initiation, an anticipatory postural adjustment (APA) phase normally precedes and accompanies the initiation of the stepping phase [27–30]. For forward stepping, these APAs involve a sequence of muscle activations and changes in the ground reaction forces (loading of the initial swing leg and unloading of the initial stance leg) that move the net center of pressure beneath the feet backward and toward the initial swing limb. This motor sequence, which ends after heel off, produces the forces and moments necessary to propel the body center of mass (COM) forward and towards the single stance limb prior to stepping.

Compared with healthy control subjects, the mediolateral (M-L) and anteroposterior (A-P) ground forces characterizing APAs in patients with PD are abnormally prolonged in duration and reduced in amplitude with a delay in the sequencing between the beginning of the APA and step onset [17, 18, 31, 32]. This delay may include abnormal pauses that disrupt the posture-movement coordination and may precipitate freezing of gait (FOG). While the APA is normally almost always present during voluntary stepping, it may often be absent in patients with PD [17, 19, 20]. In such cases, hesitation delays are readily observable. Thus, the normal spatial and temporal coordination between the APA and stepping components of gait initiation is disrupted in PD in association with start hesitation and FOG.

**Figure 1:**
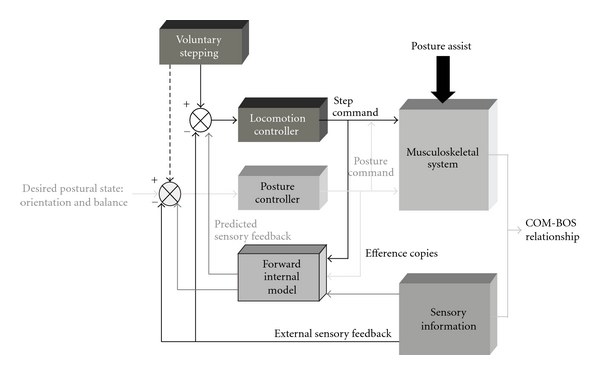
Forward internal model for posture and locomotion coupling during the initiation of gait. To initiate stepping locomotion, posture and locomotion networks are activated in parallel to generate motor commands where the posture network acts on the stepping controller. This motor output modifies the body center of mass-base of support (COM-BOS) relationship. With external posture assistance (e.g., mechanical or sensory simulation of single stance limb loading) that enhances weight transfer to the single stance limb, an efferent copy of the motor commands and sensory information about the actual state of the body can be used by the CNS to modify the two commands in advance based on an internal representation of the body and external environment (forward model). Online sensory information can also modulate posture and locomotion via external feedback.

In gait initiation, the anticipatory nature of the postural-step coordination appears to involve a role for motor prediction. A forward internal model (Figure 1) is a neural mechanism that predicts (estimates) the future state of a system given the current (actual) state and the sensorimotor control signals [33–37]. The use of a forward model for coordination between posture and locomotion could operate such that the neural circuits for initiating stepping would normally be actively delayed until the APAs that generate the weight transfer from bipedal to single leg support have achieved single stance limb loading [38, 39]. This transition in stance support reflects a change in the body center of mass-base of support (COM-BOS) relationship. Thus, using internal and external feedback information, the forward model would determine if the APAs have achieved the sufficient anticipated postural state (e.g., COM position and motion relative to the BOS) before initiating the gait cycle and finishing the postural phase.

In Figure 1, the integrated networks for posture and locomotion are activated in parallel [38–41] to generate a posture command for segmental orientation and balance and a step command. These motor outputs will modify the COM-BOS relationship. If an external mechanical or sensory event that assists with the APA by facilitating weight transfer is applied early in the postural adjustment phase, sensory information about the limb loading conditions, together with an efference copy of the motor commands sent to the forward model estimating the anticipated limb loading conditions, can be used by the CNS to modify the two commands in advance based on an internal representation of the body. Sensory information produced by movement can also be used online to modulate posture and movement via external feedback mechanisms. Conceivably, the posture assistance provided by external mechanical effects and/or sensorimotor enhancement could decrease the completion time of the weight transfer compared with the predicted time of completion without assistance and/or improve the fidelity of the information associated with the changes in limb loading reflecting postural state conditions during the APA. Based on a mismatch between the predicted and actual limb loading conditions determined from the forward internal model, the initiation release of stepping would be advanced in time and occur earlier. Reinforcement of the posture-locomotion coordination with posture-assisted locomotion (PAL) training could lead to adaptive changes in the internal model for step initiation.

The mechanisms contributing to impaired gait initiation in PD are poorly understood. It has been hypothesized that postural instability and gait dysfunction in PD result from alterations in the output of the basal ganglia to the pedunculopontine nucleus (PPN) in conjunction with the progressive degeneration of the large cholinergic neurons of this nucleus [42, 43]. The PPN has important inputs to regions of the mesencephalic extrapyramidal area and pontomedullary reticular formation that play a role in the pattern generation for locomotion and integration of posture and movement [44]. Alternatively, it has been proposed that impaired gait initiation results from dysfunction of the basal ganglia and a resulting suppression or underactivity of the supplementary motor area [45–47], a region of the frontal cortex critically involved in the planning and preparation for movement. Models of posture and movement coupling [38, 40], such as the model presented in Figure 1, often emphasize that the voluntary command to initiate movement, including the timing signal, must be integrated with brain stem and spinal centers that mediate the control of posture. Accordingly, the supplementary motor area may play a role in providing feedforward information about the internal model to both the basal ganglia and posture and locomotion control regions in the brain stem. The fact that levodopa can often improve gait initiation and locomotion in patients with off-medication impairment [48] provides evidence that alterations in basal ganglia output to both cortex and brain stem likely play a role in both the triggering of movement initiation and coupling of posture and locomotion. However, in advanced disease, posture and gait abnormalities often become resistant to levodopa replacement therapy, suggesting that the progressive degeneration or dysfunction of nondopaminergic regions of the neuraxis [49], such as the PPN, becomes the principal pathology that mediates the disordered coupling between posture and locomotion.

In PD, difficulties with achieving the postural prerequisites for stepping could contribute to gait initiation delays, “start hesitation,” and FOG. With postural assistance, the usually prolonged APA duration and reduced amplitude accompanying PD could be, respectively, shortened and increased to enhance posture requirements and allow an earlier step onset time. Thus, the rationale for the PAL training approach is that the expected limb loading conditions associated with weight transfer to the single stance limb are enhanced (e.g., achieved earlier and more effectively) compared with what would usually be expected without the assistance. If patients with PD retain the capacity to adapt their putative internal model for stepping with PAL training, then it might be possible to remodel the timing sequence and other characteristics of posture and locomotion components of gait initiation.

## 3. Experimental Support

### 3.1. Postural Assistance with Weight Transfer Acutely Enhances Posture and Locomotion Coupling and Performance during the Initiation of Stepping

**Figure 2:**
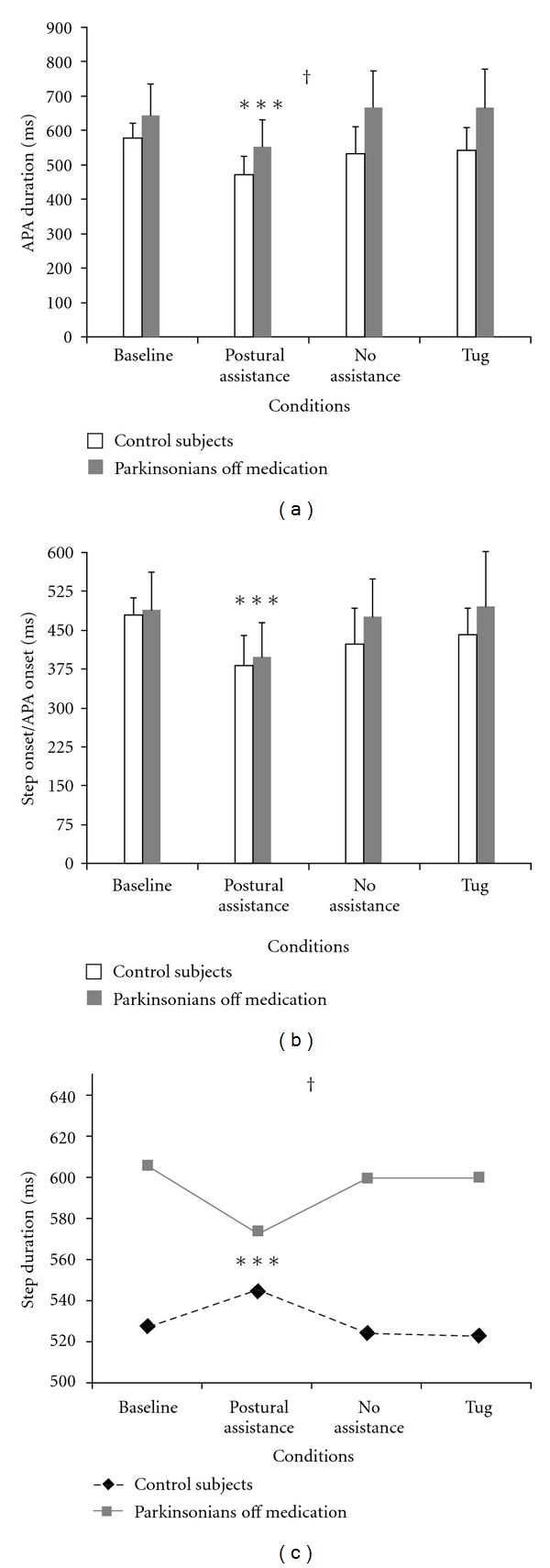
The group mean values plus 1 SD for (a) APA duration, (b) first-step onset time relative to APA onset, and (c) first-step duration in control subjects (CS: white bars) and subjects with Parkinson's disease off medication (off: gray bars). The four experimental conditions are initial baseline trials without postural assistance (Baseline), trials with lateral postural assistance (Postural assistance), follow-up trials without postural assistance (No assistance), and trials with a mechanical tug that provided no direct postural assistance (Tug). Data from [18]. ^†^Significant differences between groups. ***Significant difference between the postural assistance condition (ASSIST) and the others.

A first study examined the influence of a lateral postural assist on step initiation in patients with PD and healthy controls [18]. Subjects performed self-paced rapid forward steps. In one condition, the APA was assisted shortly after onset (i.e., triggered by a 5% change in loading force from baseline beneath the initial swing leg) with a lateral pull applied to the pelvis (toward the initial stance side) by a motor-driven robotic system. Ground reaction forces and whole body kinematics were recorded to characterize the APA (extracted from the mediolateral center of pressure displacement) and step characteristics (derived from the first stepping leg ankle marker displacement). Overall, persons with PD (Hoehn and Yahr stage mean = 2.0) [50] tested off anti-parkinsonian medications had a longer APA duration and longer first-step duration than control subjects. With the postural assistance, the APA duration for both groups was shorter, the step onset time relative to the APA onset was earlier, and the speed of the first step became faster (i.e., step duration decreased while step length did not change) for PD subjects (Figure 2). These improvements in stepping performance could be related to the influence of a sensory cue provided by the waist-pull stimuli. This possibility was assessed in a tug condition that was delivered in the same way as the posture assist but involved a displacement that was reduced to 25% of the assist waist-pull. The tug resulted in a stimulus that gave very little mechanical assistance with the lateral weight transfer but provided a vigorous stimulus to the pelvic area that could conceivably have been used as a timing cue to facilitate stepping. No changes in performance from baseline were observed when a tug stimulus cue was presented (Figure 2). This ruled out that the posture assist was attributable to sensory cueing. It is also possible that stepping practice alone could have accounted for the findings. A separate practice group is needed to definitively account for this possibility. However, the fact that a block of trials without mechanical assistance or sensory cues was always presented either as the second to last block or last block of trials and that these trials did not differ from the initial baseline for any of the measurements provides evidence that the effects of the postural assist could not be attributed to practice alone.

These findings indicated that rapid step initiation could be acutely enhanced through external assistance that facilitated weight transfer and thereby modified posture and locomotion coupling in individuals with early stage PD while off of their anti-parkinsonian medication as well as in healthy older people. In addition to the mechanical effects of the robotic assistance that contributed to passively shortening the APA duration and first-step onset timing, the neural circuits for initiating stepping could have been actively triggered and modified in interaction with the enhanced sensory feedback providing information about the expected or actual state conditions (e.g., center of mass position and motion relative to base of support) associated with the evolving APA [38] (Figure 1).

Applying assistive mechanical displacement laterally at the pelvis indirectly modifies the loading forces beneath the feet that influence sensory inputs for posture and gait control [51]. Therefore, it is conceivable that if loading force information is important for timing the release of the gait cycle and other locomotion characteristics, then APA and step parameters should also be modifiable by directly manipulating the loading forces during gait initiation. Alterations in limb loading may also be important because of past work demonstrating that patients with PD may show abnormalities in load receptor-mediated proprioception during stance and gait [52]. Moreover, if limb loading information is important for the control of step initiation as in ongoing gait, then healthy individuals would also be expected to demonstrate modifications in stepping when limb load input is perturbed. Hence, we have extended our waist-pull posture-assisted locomotion approach by developing a controllable, vertical dropping-elevation perturbation system to induce changes in posture and locomotion coupling [53].

**Figure 3:**
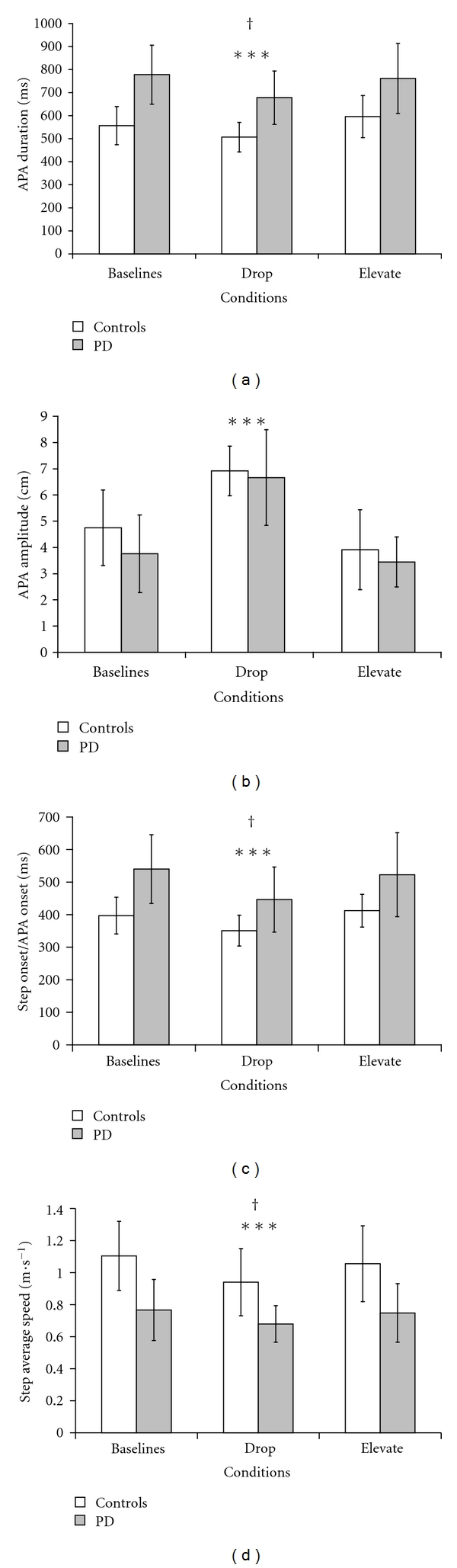
The group mean values (±1 SD) for patients with Parkinson's disease (PD) on medication (gray bars) and healthy control subjects (white bars) during rapid self-paced step initiation under the different experimental conditions are presented for (a) APA duration, (b) APA amplitude, (c) step onset relative to the APA onset, and (d) first-step speed. Data from [53]. ^†^Significant differences between groups. ***Significant difference between the postural assistance condition (DROP) and the other two.

Eight patients with PD (modified Hoehn and Yahr Stage score 2.5 to 3.0) [50] and eight healthy control subjects performed rapid self-triggered step initiation with the impending single stance limb positioned over a pneumatically actuated platform. All subjects had been experiencing start hesitation or FOG. In perturbation trials, the APA was either assisted by moving the stance limb ground support surface vertically downward (DROP) or resisted by moving it upward (ELEVATE), shortly after the onset of the APA phase. Overall, patients with PD demonstrated a longer APA duration, longer time to first-step onset, and slower step speed than controls. In both groups, the DROP of the stance limb reinforced the intended APA kinetic changes for lateral weight transfer (i.e., significant reduction in APA duration and increase in peak amplitude) and resulted in positive changes in step characteristics (i.e., earlier time to first-step onset and faster step) compared with other conditions (Figure 3). In contrast, during ELEVATE trials that opposed the intended weight transfer forces, both groups rapidly adapted their stepping to preserve standing stability to the detriment of step characteristics by decreasing step length and duration and increasing step height and foot placement laterally. These findings suggest that sensory information associated with limb load and/or foot pressure occurring prior to the release of stepping modulates the spatial and temporal parameters of posture and locomotion in interaction with a centrally generated feed-forward mode of neural control. Moreover, impaired step initiation in PD may at least acutely be enhanced by augmenting the coupling between posture and locomotion through changes in limb load proprioception.

### 3.2. Training-Induced Changes in Postural and Locomotion Coupling and Performance during Step Initiation

From a rehabilitation standpoint, it would be important to know whether longer-term changes in posture and locomotion coupling are achievable with training. It is generally acknowledged that patients with PD can improve their motor performance through practice training, but that they may achieve less improvement and take longer to change their performance than healthy adults [54, 55]. Thus, it is conceivable that posture-assisted training could be applied to adaptively remodel the coupling between posture and locomotion in PD. We have recently completed a feasibility intervention study aimed at determining the effects of PAL training using mechanosensory limb load assistance (i.e., drop of support surface on single stance side) compared with sensory enhancement of weight transfer (i.e., vibration of support surface on single stance side) on posture and locomotion coupling and performance during step initiation in patients with PD.

Seven subjects (mean age = 72.9 years) with moderate PD (modified Hoehn and Yahr Stage score 2.5 to 3.0) [50] and on medications received baseline testing followed by twice weekly PAL training for six weeks. For each training session, the drop assist group performed 50 self-initiated rapid stepping trials where the stance limb ground support surface was moved vertically downward by 1.5 cm over 100 ms shortly after the onset of the APA phase (change in single stance limb load vertical force by 5% from baseline standing) similar to our earlier study [53].

A second group (mean age = 75.3 years) consisted of eight subjects with moderate PD (modified Hoehn and Yahr Stage score 2 to 3) [50] received vibration assist training through mechanical vibration stimulus (200 Hz over 100 ms) of the single stance side support surface applied at the same relative time point during the early APA phase as the stimulus for the drop assist group. These PD subjects were tested on medications and followed the same testing and training schedule as the drop assist group.

**Figure 4:**
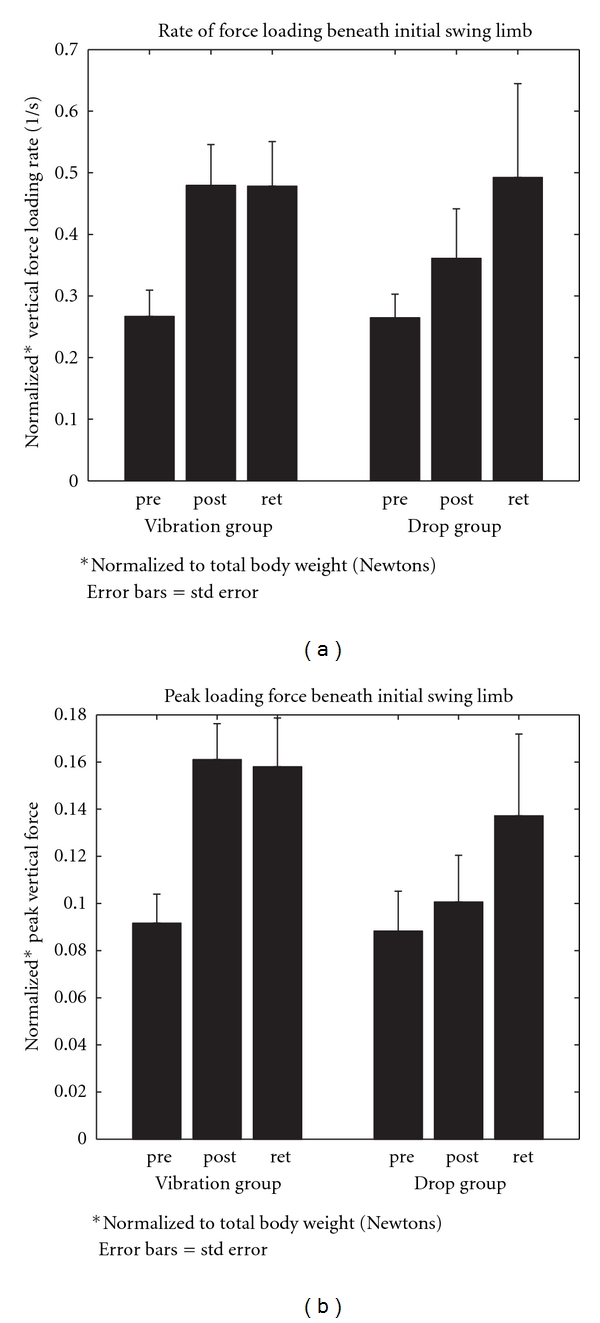
The group mean values plus 1 SEM for initial swing limb APA (a) rate of loading force and (b) peak loading force amplitude measured at baseline prior to posture assist locomotion (PAL) training (pre), immediately after training (post), and six weeks after the completion of training (ret) PD subjects in drop assist and vibration assist training groups. Unpublished data.

**Figure 5:**
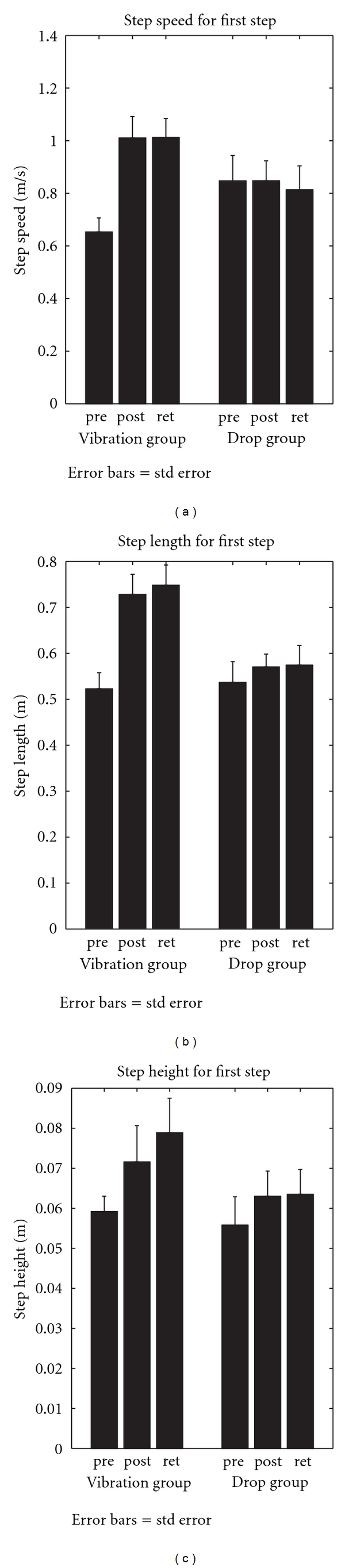
The group mean values plus 1 SEM for first-step (a) speed, (b) length, and (c) height measured at baseline prior to posture-assisted locomotion (PAL) training (pre), immediately after training (post), and six weeks after the completion of training (ret) PD subjects in drop assist and vibration assist training groups. Unpublished data.

Immediate posttesting completed after the six-week training phase indicated several training-associated improvements in kinetic APA and stepping kinematic variables. First, for APA characteristics (Figure 4), both the rate and peak amplitude of the loading force beneath the initial swing limb for lateral weight transfer prior to stepping were, respectively, significantly increased, by 53% and 44% across both training groups. Follow-up testing occurring six weeks after the completion of training showed that these increases were retained. Second, significant group by time of testing interactions for first-step kinematic data (Figure 5) showed that both step speed and length were, respectively, increased by 54% and 38% for the vibration assisted group between the baseline and immediate posttest and remained greater at retention testing. First-step height (Figure 5) was also increased by 17%–25% for both groups between pretesting and both posttesting periods.

Although systematic investigation of the accuracy of the following observations has yet to be addressed, two aspects of the approaches appear to be important for successful implementation. First, the triggering of the posture enhancement stimulus should be activated by the subject's self-initiated postural action, and, second, the time of delivery of the event should occur shortly after the onset of the posture event. This self-triggered and early event timing might enable the external information to be incorporated into the forward control of the posture and locomotion sequence.

## 4. Implementation of Posture-Assisted Locomotion Rehabilitation

### 4.1. Targeting Posture and Locomotion Coupling in the Rehabilitation of People with Parkinson's Disease

Better understanding of posture and locomotion coupling problems has significant relevance for physical therapy practice. To date, there has been a lack of interventions to directly address posture and locomotion coupling problems. Interventions such as PAL hold promise for specifically enhancing or assisting with the posture requirements that precede and accompany locomotion and other movements in order to improve the spatial and temporal coupling. Ultimately, the goal is to enhance posture and locomotion coupling to improve performance in functional activities, foster greater quality of life, and decrease fall risk.

Two recent reviews [56, 57] point out that while mounting progress has been made with providing some evidence for the effectiveness of current exercise rehabilitation approaches on balance and gait outcomes in PD, considerable gaps remain in the evidence base for specific interventions and in identifying the optimal content of exercise interventions. Part of the challenge in effectively addressing these gaps in knowledge is in formulating conceptual and theoretical frameworks and models that take into account the complexities or influential factors. Greater focus on the ways that the interrelationship or coupling between posture, balance, and locomotion elements advantage and constrain functional performance would appear to be one such area where rethinking the framework for intervention development may be useful for advancing clinical practice.

### 4.2. Expanding the Application of the PAL Model

As mentioned previously, the difficulties with performing complex whole body posture and locomotion sequences in PD have been observed for a range of different functional activities. For example, significant timing delays that have been identified for the sequencing between the anticipatory forward weight transfer phase and the intended vertical ascent phase of sit-to-stand [24] or between anticipatory segmental body rotations that steer the COM prior to foot redirection in gait turning have been observed in PD patients [23]. These temporal disruptions are very analogous to the temporal disruption of posture and locomotion coupling seen for gait initiation. Application of the PAL approach through mechanical and/or sensory enhancement of the early postural phase may trigger an earlier release of subsequent movement and possible enhancement of overall performance. Improvement in stepping patterns using vibratory sensory stimulation of the trunk postural muscles during ongoing walking in persons with PD and healthy controls, as demonstrated by De Nunzio et al. [16], provides a promising example of posture-locomotion coupling applicable to intervention. Impairments in the interaction between posture and whole body movement tasks will need further investigation to support the hypothesized view of impaired coupling of posture and goal-intended components of action in individuals with PD.

## 5. Summary

In this perspective and review, we have advanced the view point that approaches to rehabilitation interventions that focus on changing separate isolated components for posture, balance, and gait in persons with PD may have limited effectiveness due to the importance of posture and locomotion coupling. Alternatively, there is neurophysiological and experimental support for the idea that posture and locomotion are highly integrated components of action that require understanding of how these control functions are interactively coupled. Moreover, there is evidence to indicate that individuals with PD have particular problems with coupling or sequencing posture and locomotion during complex whole body movements that are associated with falls. Expanding or shifting current conceptual and theoretical models of rehabilitation beyond posture/balance and gait-centered intervention focuses by incorporating posture and locomotion coupling problems as a target for rehabilitation outcomes may help to optimize and improve the effectiveness of current clinical practice in this important area of rehabilitation for persons with PD.
